# Urinary interleukin-1β levels among gynecological patients

**DOI:** 10.1186/s13048-014-0104-4

**Published:** 2014-11-18

**Authors:** Kamisha T Woolery, Mitchel S Hoffman, Joshua Kraft, Santo V Nicosia, Ambuj Kumar, Patricia A Kruk

**Affiliations:** Department of Pathology and Cell Biology, University of South Florida, 12901 Bruce B. Downs Blvd., Tampa, FL 33612 USA; Department of Obstetrics and Gynecology, University of South Florida, Tampa, FL 33612 USA; Department of Internal Medicine, University of South Florida, Tampa, FL 33612 USA; H. Lee Moffitt Cancer Center, Tampa, FL 33612 USA

**Keywords:** Interleukin-1 beta, Obesity, Ovarian cancer, Urine, Body mass index

## Abstract

**Background:**

Early detection of epithelial ovarian cancer (OC) is necessary to overcome the high mortality rate of late stage diagnosis; and, examining the molecular changes that occur at early disease onset may provide new strategies for OC detection. Since the deregulation of inflammatory mediators can contribute to OC development, the purpose of this pilot study was to determine whether elevated urinary levels of Interleukin-1beta (IL-1 beta) are associated with OC and associated clinical parameters.

**Methods:**

Urinary and serum levels of IL-1 beta were analyzed by ELISA from a patient cohort consisting of healthy women (N = 10), women with ovarian benign disease (N = 23), women with OC (N = 32), women with other benign gynecological conditions (N = 22), and women with other gynecological cancers (N = 6).

**Results:**

Average urinary IL-1 beta levels tended to be elevated in ovarian benign (1.26 pg/ml) and OC (1.57 pg/ml) patient samples compared to healthy individuals (0.36 pg/ml). Among patients with benign disease, urinary IL-1β levels were statistically higher in patients with benign inflammatory gynecologic disease compared to patients with non-inflammatory benign disease. Interestingly, urinary IL-1 beta levels tended to be 3-6x greater in patients with benign ovarian disease or OC as well as with a concomitant family history of ovarian and/or breast cancer compared to similar patients without a family history of ovarian and/or breast cancer. Lastly, there was a pattern of increased urinary IL-1 beta with increasing body mass index (BMI); patients with a normal BMI averaged urinary IL-1 beta levels of 0.92 pg/ml, overweight BMI averaged urinary IL-1 beta levels of 1.72 pg/ml, and obese BMI averaged urinary IL-1 beta levels of 5.26 pg/ml.

**Conclusions:**

This pilot study revealed that urinary levels of IL-1 beta are elevated in patients with epithelial OC supporting the thought that inflammation might be associated with cancer progression. Consequently, further studies of urinary IL-1 beta and the identification of an inflammatory profile specific to OC development may be beneficial to reduce the mortality associated with this disease.

## Background

Ovarian cancer (OC) is the fifth leading cause of cancer death among women after lung, breast, colorectal, and pancreatic cancer [1]. OC is highly treatable when diagnosed in the early stages; however, the majority of cases are diagnosed in late stage and the 5-year survival is approximately 27% [1]. In order to detect early stages of OC, there is a need for a better understanding of the etiology of this disease as well as improved screening options. Currently, there are three screening procedures in use for OC detection: bimanual pelvic examination, serum CA125, and transvaginal ultrasonography (TVS) [2]. Pelvic examinations are not effective in distinguishing a premalignant lesion from a normal ovary [3]. Serum CA125 is elevated in 47% of women with early stage OC and elevated in 80-90% of advanced stage OCs [4]. However, CA125 can also be elevated in healthy women and in patients with benign ovarian disease [5]; as well as, in other cancers such as endometrial, pancreatic, lung, breast, colorectal, and certain gastrointestinal tumors [6]. The pairing of TVS with serum CA125 improves OC diagnosis; however, sonography can result in false positive results and unnecessary surgery [7]. Unfortunately, utilizing these methods either alone or in combination does not produce the desired results for early disease detection. Therefore, examining the molecular changes that occur at early disease onset may provide new approaches or biomarkers for OC detection.

Deregulation of inflammation due to overexpression of proinflammatory proteins contributes to the malignant phenotype by supporting cancer cell growth and tumor invasion. Since the proinflammatory cytokine, interleukin-1β (IL-1β), is constitutively expressed in OC [8] and elevated in serum of OC patients [9], in this pilot study, we sought to assess whether elevated urinary levels of IL-1β are associated with OC and related clinical parameters.

## Methods

### Patient cohort

With prior University of South Florida Institutional Review Board committee approval for study # 4739, urine and serum samples were collected from an initial cohort of healthy controls (N = 7), women with ovarian benign disorders (N = 12), and patients with epithelial OC (N = 20) at the H. Lee Moffitt Cancer Center (MCC). The second urine and serum cohort collected with University of South Florida Institutional Review Board committee approval for study # Pro00003119 at the University of South Florida (USF) consisted of healthy controls (N = 3), women with ovarian benign disease (N = 11), women with epithelial OC (N = 12), women with other benign gynecologic disease (N = 22), and other gynecologic cancers (N = 6). The OC category, diagnosed at time of initial cytoreductive surgery, consisted of women diagnosed with OC and primary peritoneal cancer, which is often related to OC. The samples collected from women with ovarian benign disease consisted of a broad range of non-malignant gynecologic disorders. Though these 93 samples comprise a small pilot study (Table 1), they are representative of a typical clinical practice with regards to histological distribution. Table 1 shows an age matched subset (N = 87) developed from the OC patients in the USF + MCC cohorts to produce an OC patient group with an age range (26–75 years) within two standard deviations of mean age to match healthy control (37–60 years) and ovarian benign disease (28–81) groups, respectively. Where possible, H & E sections from paraffin blocks were reviewed to confirm the histologic diagnosis according to International Federation of Gynecology and Obstetrics (FIGO) scores. H & E sections were also reviewed among patients with benign disease to distinguish between patients with inflammatory disease (including endometriosis, pelvic inflammatory disease) and non-inflammatory disease (including cystademona, leiomyoma, ovarian cysts). Anonymized information regarding patient age, body mass index (BMI), and tumor type were also obtained as per availability.

**Table 1:** **Histologic diagnoses and clinical characteristics of the study cohort**

	**Age (mean ± SD)**	**Age (range)**
USF + MCC cohort		
**Normal (10)**	49.3 ± 10.3	37-66
**Benign (23)**	52.68 ± 17.7	28-81
Serous cystadenoma (8)		
Mucinous cystadenoma (3)		
Benign ovarian tumor (2)		
Cyst (2)		
Endometrioma (2)		
Polycystic disease (2)		
Cystadenomofibroma (1)		
Leiomyoma (1)		
Ovarian fibroma (1)		
Thecoma (1)		
**Other benign gynecological conditions (22)**	42.59 ± 9.5	30-62
**Other gynecological cancers (6)**	59.17 ± 8.2	20-77
**Ovarian cancer (32)**	60.91 ± 15.6	26-92
Serous (27)		
Mucinous (4)		
Endometrioid (1)		
**Age match (26)**	57.7 ± 15.0	26-78
Serous (22)		
Mucinous (3)		
Endometrioid (1)		

### Sample preparation

Urine and serum samples were collected from patients, anonymized to protect patient identity, and released from the tissue banks for this research project. All samples were kept on ice following collection. MCC urine samples were treated with a standard protease inhibitor cocktail (80 ug/mL 4-(2 aminoethyl)-benzene sulfonyl fluoride, 200 ug/mL ethylenediaminetetraacetic acid (EDTA), 0.2 ug/mL leupeptin, 0.2 ug/mL pepstatin, Sigma Scientific, St. Louis, MI). All urine samples were centrifuged at 3000 × g. Urinary supernates and serum samples were then aliquoted and stored at −20°C.

### Enzyme-linked immunosorbant assay

To measure IL-1β levels in patients’ urine and serum, samples were assayed using the quantitative sandwich enzyme-linked immunosorbant assay (ELISA; R&D Systems, Inc., Minneapolis, MN) according to the manufacturer’s instructions. Fluorescence was read on an ELx800 Absorbance Microplate Reader (Biotek, Winooski, Vermont) using Gen5 Data Analysis Software (Biotek, Winooski, Vermont). Resultant values were derived from a standard curve and expressed as the mean IL-1β concentration of triplicate samples ± standard error (S.E.).

### Statistical analysis

Samples for IL-1β ELISA were run in triplicate and the data subjected to descriptive, parametric, Kruskal-Wallis, Mann–Whitney U, Spearman correlation, and Wilcoxon W analyses.

## Results

### Urinary levels of IL-1β generally decrease with increasing patient age

**Figure 1:**
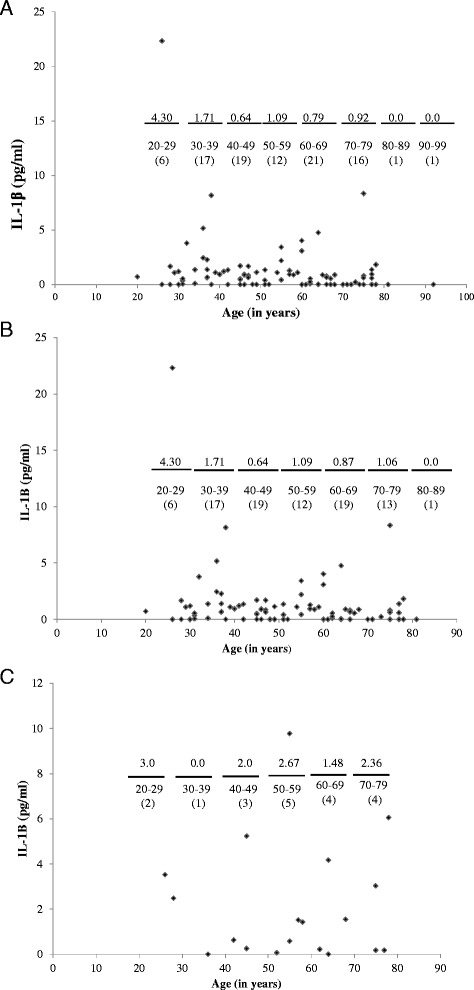
**Urinary and serum levels of IL-1** **β** **are elevated in 20–29 and 30–39 years age groups.** ELISA was utilized to measure IL-1β (mean pg/ml) in the urine and serum of study samples. **(A)** Urinary levels in all samples (N = 92). **(B)** Urinary levels in age matched subset (N = 86). **(C)** Serum levels in available samples. Samples were examined in triplicate and the data expressed as mean IL-1β levels (pg/ml) per age decade (mean value noted above bar depicting age decades with number of patients/decade category indicated in parenthesis) following descriptive, parametric and Kruskal-Wallis analyses.

Levels of urinary IL-1β were compared in 93 patients of the entire cohort and 87 patients of the age matched subset. Though this cohort comprises a small pilot study, it is representative of our institutional clinical practice in regard to OC histology and stage distribution as well as in keeping with other comparable pilot studies. Average urinary IL-1β levels were compared to patient age and as divided by decade grouping. The highest levels of urinary IL-1β were found in the 20–29 and 30–39 age groups in both the entire cohort (Figure 1A) and the age matched subset (Figure 1B), respectively, suggesting an inverse relationship between increasing age and urinary IL-1β levels. Additionally, levels of serum IL-1β were compared in 19 patients of the entire cohort. In agreement with comparisons between urinary IL-1β levels and age, the highest average serum IL-1β level was detected in the 20–29 age group (Figure 1C), further suggesting a possible inverse correlation between age and IL-1β levels. Despite the tendency for increased urinary IL-1β levels in the 20–29 and 30–39 age groups, no statistical differences were found.

### Urinary IL-1β levels appear elevated in patients with ovarian cancer

**Figure 2:**
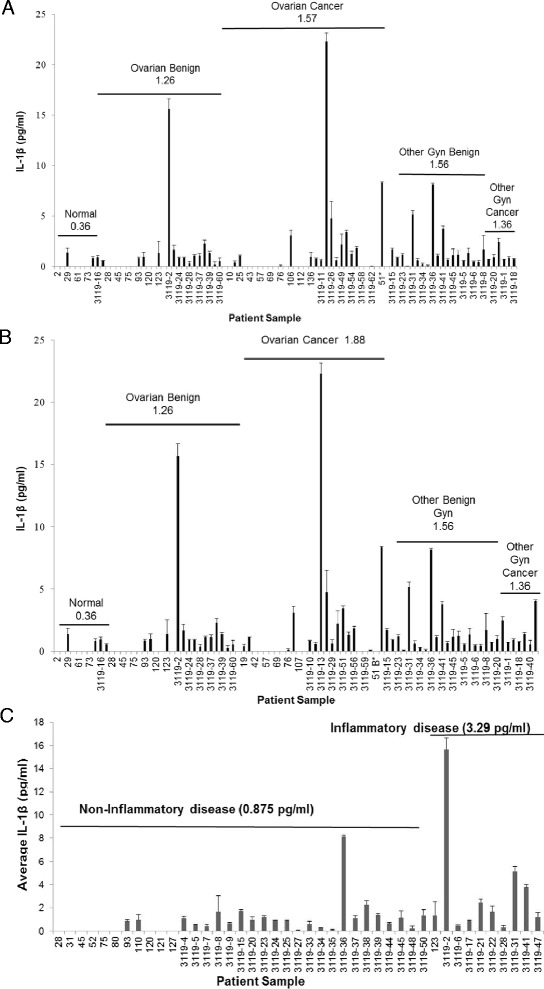
**Urinary IL-1β** **levels are elevated in ovarian cancer patients.** Urinary samples were analyzed in triplicate by ELISA and data expressed as mean in pg/ml noted above bar depicting clinical diagnostic category following descriptive, Mann–Whitney U and Wilcoxon W analyses. **(A)** Entire cohort of healthy controls, benign ovarian disorders, ovarian cancer patients, other gynecological benign disorders, and other gynecological cancers; **(B)** age matched subset of healthy controls, benign ovarian disorders, ovarian cancer patients, other gynecological benign disorders, and other gynecological cancers. **(C)** Non-inflammatory benign disorders and inflammatory benign disorders in all benign ovarian and other benign gynecological disorders.

The levels of urinary IL-1β were generally negligible (average 0.36 pg/ml) in healthy control samples of the entire cohort and the age matched subset, respectively (Figure 2A and B). However, urinary IL-1β levels from women with benign ovarian disease were approximately 4-fold higher compared to healthy controls (average benign 1.26 pg/ml) (Figure 2A and B). Urinary levels of IL-1β from OC patients exhibited the highest average levels of 1.57 pg/ml IL-1β in the entire cohort (Figure 2A) and 1.88 pg/ml IL-1β in the age matched subset (Figure 2B), respectively. Further, levels of urinary IL-1β in non-ovarian benign gynecological disorders (average 1.56 pg/ml) and in non-ovarian gynecological cancers (average 1.36 pg/ml) were statistically increased compared to healthy controls (p ≤0.005 and 0.05, respectively) (Figure 2A and B). Lastly, when urinary IL-1β levels were compared among patients with benign disease, but differentiated on the basis of noted inflammatory involvement (including endometriosis, pelvic inflammatory disease), average urinary IL-1β levels were statistically increased in inflammatory benign disorders (1.88 pg/ml) compared to non-inflammatory benign disorders (0.82 pg/ml) (p =0.001) (Figure 2C).

**Figure 3:**
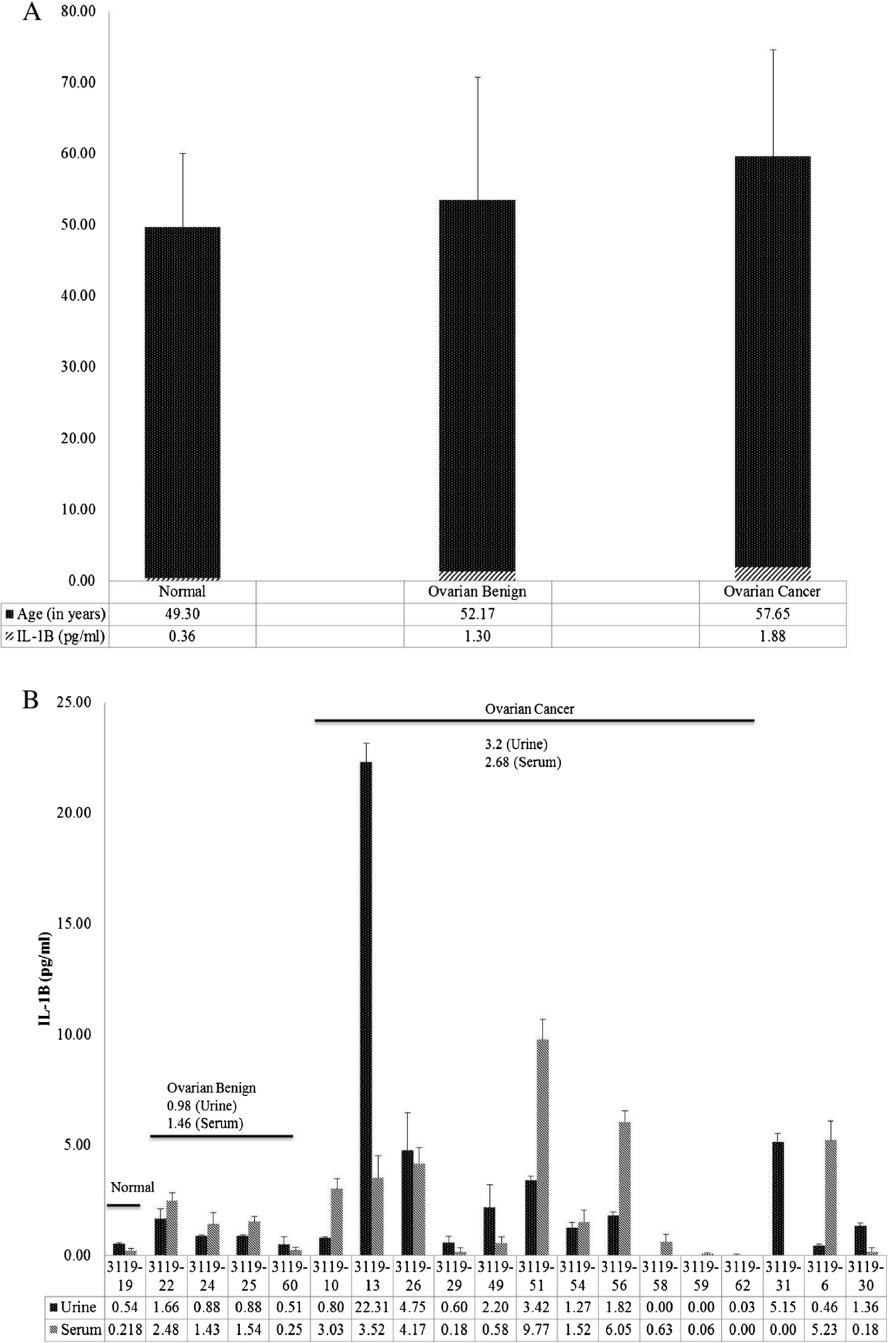
**Urinary and serum IL-1β** **levels are elevated in ovarian cancer patients.** IL-1β levels were measured and compared by **(A)** average age and average level urinary IL-1β (pg/ml) per diagnosis of healthy (normal) (N = 10), benign ovarian disorder (N = 25), and ovarian cancer (N = 27) or measured from **(B)** paired urinary and serum samples from normal, ovarian benign, and ovarian cancer patients. Samples were analyzed in triplicate by ELISA and data expressed as mean (pg/ml) following descriptive, Mann–Whitney U and Wilcoxon W analyses **(A, B)** ± standard deviation of age in years **(A)**.

When urinary IL-1β levels were compiled in the age matched cohort with respect to diagnosis and age, the average age in the healthy controls, benign ovarian disorders, and OC patients was 49.3, 52.2, and 57.7 years, respectively (Figure 3A). The average urinary levels of IL-1β in these healthy controls, benign ovarian disorders, and OC patients was 0.36 pg/ml, 1.30 pg/ml, and 1.88 pg/ml, respectively (Figure 3A) supporting a trend for increased urinary IL-1β levels with disease progression.

Levels of corresponding urine and serum IL-1β were available from 19 patients. Compared to controls, paired serum IL-1β showed a similar pattern of increased average IL-1β levels in the benign ovarian and OC group, respectively, with the highest average levels in the OC group (Figure 3B). Since there was a significant Spearman correlation between serum and urinary IL-1β levels in ovarian benign disorders (r = 0.949, N = 4, p = 0.051) and in ovarian cancers (r = 0.724, N = 11, p = 0.012), and there was a very limited number of available serum samples, all remaining clinical comparisons were completed in urine samples only.

### Urinary IL-1β levels are increased in patients with a family history of cancer

**Figure 4:**
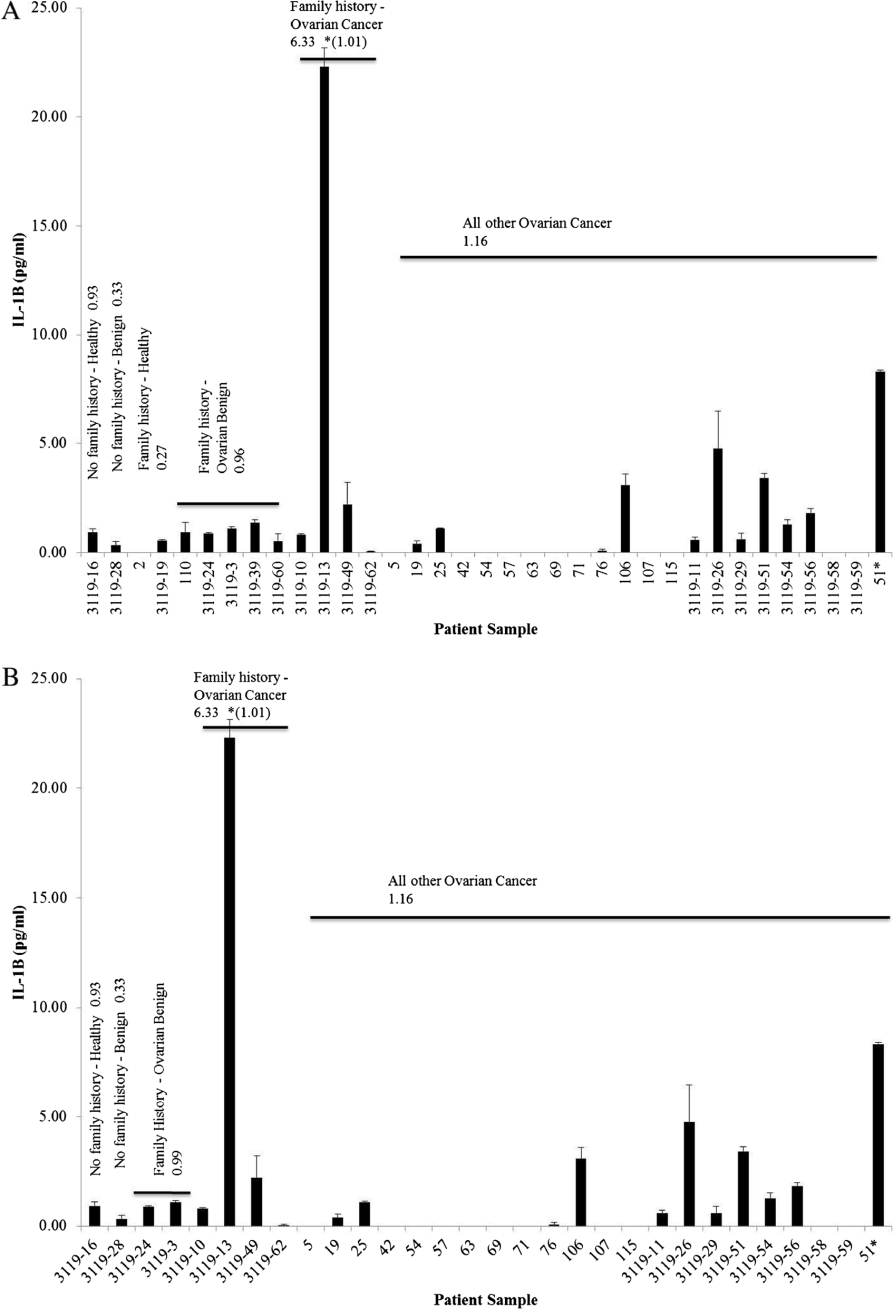
**Urinary IL-1β** **levels increase with familial history of cancer.** Urinary IL-1β was analyzed in triplicate by ELISA and data expressed as mean (pg/ml) ± standard error in age matched subset cohort following descriptive, Mann–Whitney U and Wilcoxon W analyses. **(A)** Patients with family history of any cancer. **(B)** Patients with family history of ovarian and/or breast cancer in first degree family members only.

Using the age matched cohort subset, urinary IL-1β levels were analyzed with respect to a family history of cancer though the BRCA1 status of these patients could not be confirmed. While the number of specimens was small, in a patient with benign ovarian disease and no known family history of cancer, urinary IL-1β levels were 0.33 pg/ml; while average levels were 0.96 pg/ml in five patients with benign ovarian disease and a family history of cancer (Figure 4A). Though we could not identify the lack of family history of cancer in most of the OC patients, in those OC patients with confirmed family history of cancer, average urinary IL-1β levels were 6.33 pg/ml (Figure 4A), while the average urinary IL-1β levels of the remaining OC patients was 1.16 pg/ml. However, when sample 3119–13 which was about 20-fold higher than other samples in the OC group was excluded from the analyses, the average urinary IL-1β levels decreased to 1.01 pg/ml (Figure 4A).

Thirteen patient samples of the age matched subset were further narrowed to 8 patients with a first degree family history of ovarian and/or breast cancer. In two patients with benign ovarian disease and a family history of ovarian and/or breast cancer, average urinary IL-1β levels were three times higher (0.99 pg/ml) compared to patients with benign ovarian disease, but without a family history of ovarian and/or breast cancer (0.33 pg/ml). Likewise, among three patients with OC and a family history of ovarian and/or breast cancer, average urinary IL-1β levels were elevated (6.33 pg/ml) compared to OC patients without a family history of ovarian and/or breast cancer (1.16 pg/ml) (Figure 4B).

### Urinary IL-1β levels correlate with body mass index (BMI)

**Figure 5:**
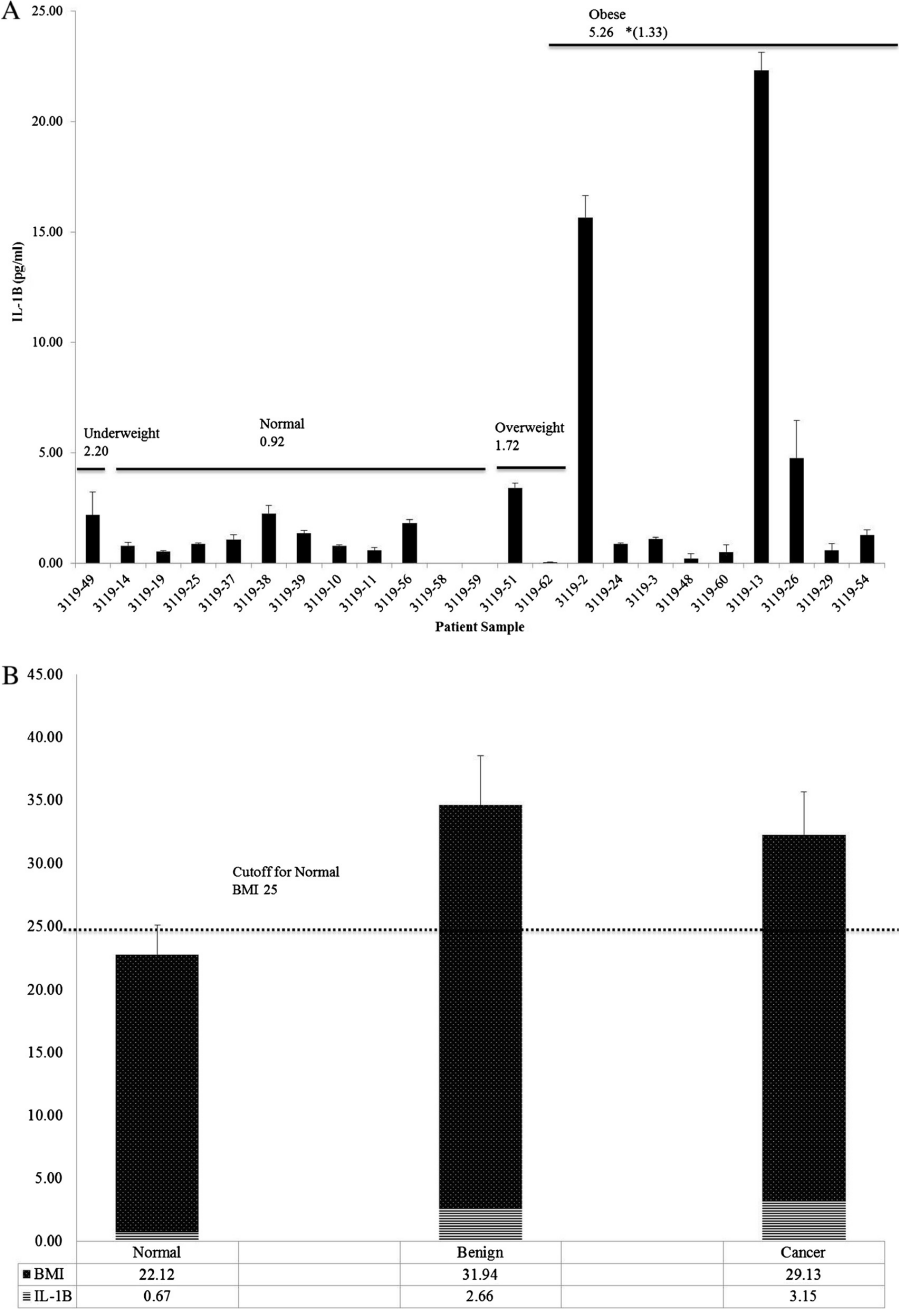
**Urinary IL-1β** **levels are elevated in overweight and obese patients. (A)** Urinary IL-1β levels in patient samples in relationship to BMI: Underweight (N = 1), Normal (N = 11), Overweight (N = 2), Obese (N = 9). Urinary IL-1β was analyzed in triplicate by ELISA and data expressed as mean (pg/ml) ± standard error in the age matched subset cohort. **(B)** Average urinary IL-1β levels and average BMI per diagnosis category: Normal (N = 2), Ovarian Benign (N = 9), Ovarian Cancer (N = 11). Data expressed as mean (pg/ml) ± standard error of BMI and mean urinary IL-1β in age matched subset cohort following descriptive, Mann–Whitney U and Wilcoxon W analyses.

Urinary IL-1β levels were measured in 23 patient samples of the age matched subset and compared with their BMI. The classifications of BMI in kg/m^2^ as underweight (≤18.5), normal (18.5-24.99), overweight (25–29.99), and obese (>30) were employed according to World Health Organization standards [10]. Patients with a normal BMI had the lowest average urinary IL-1β levels of 0.92 pg/ml while overweight patients had average urinary IL-1β levels of 1.72 pg/ml, about double that found in normal BMI patients (Figure 5A). Obese patients had the highest average urinary IL-1β levels of 5.26 pg/ml; however, when samples 3119–2 and 3119–13 were excluded since IL-1β levels were at least 15-fold higher than other samples in the obese group, urinary IL-1β levels averaged 1.33 pg/ml, comparable to overweight patients though still greater than patients with normal BMI (Figure 5A). While a single patient classified as underweight had a urinary IL-1β level of 2.20 pg/ml (Figure 5A), overall urinary IL-1β levels appeared to increase with increasing BMI.

Further analyses of urinary IL-1β levels with BMI and clinical diagnosis revealed that healthy control patients had an average normal BMI of 22.12 and the lowest average urinary IL-1β level of 0.67 pg/ml (Figure 5B). Patients with benign ovarian disease fell within the obese BMI classification (31.94) and demonstrated a 4-fold higher average urinary IL-1β level of 2.66 pg/ml (Figure 5B) compared to the healthy controls. OC patients had an average overweight BMI of 29.13 and 4.7-fold higher average urinary IL-1β level of 3.15 pg/ml (Figure 3.5B) compared to the control group. While these comparisons failed to achieve statistical significance, overall, our data suggest a trend of increased urinary IL-1β levels with increasing BMI.

## Discussion

While inflammation is an essential biological process for normal development and tissue homeostasis, it is also involved in a number of pathologic conditions including tissue injury, chronic inflammation, immunological diseases, and cancer [11]. Epidemiological studies have shown a link between chronic inflammation and risk for cancer as evidenced by prolonged infection with *Helicobacter pylori* and gastric cancer, inflammatory bowel disease and colon cancer, and prostatitis and prostate cancer [12]. In the ovary, chronic inflammation resulting from repeated ovulatory wounding and repair promotes oxidative stress which enhances DNA replication errors and ultimately, oncogenesis [13]. Inflammation is regulated by several factors that can either promote or inhibit inflammation and since epithelial OCs are highly inflammatory, this pilot study evaluated urinary levels of the proinflammatory mediator, IL-1β against clinical parameters in order to gain a better understanding of this disease. This study was able to evaluate four clinical parameters in relation to urinary IL-1β levels: (1) patient age, (2) diagnosis, (3) family history of cancer, and (4) BMI.

When we considered patient age at the time of sample collection, the highest levels of urinary IL-1β levels were found in the 20–29 and 30–39 years age groups followed by declining IL-1β levels as age increased. In keeping with our findings and according to the American Society for Reproductive Medicine, a woman’s potential reproductive capacity begins to gradually decline at approximately 32 years of age and more rapidly decreases after 37 years of age [14]. IL-1β has been suggested to play a role in female reproduction; specifically in ovulation and oocyte maturation, and inflammatory-linked mechanisms, such as production and activation of proteolytic enzymes, prostaglandin production, nitric oxide production, cellular metabolism, and steroidogenesis [15,16]. Therefore, it seems likely that after 40 years of age, there would be a decrease in urinary IL-1β levels as oocyte maturation and ovulation decrease in preparation for menopause. In contrast, Vural, et al. found higher plasma levels of IL-1β in postmenopausal (≥48.6 years) women than in premenopausal (30.5 ± 2.5 years) women with levels of IL-1β decreasing below the premenopausal levels only after hormone replacement therapy [17]. Therefore, it is possible that the sharp decrease in urinary IL-1β levels seen in our study in women >60 years may be due, in part, to the usage of hormone replacement therapy; however, this clinical information was not available for confirmation.

Urinary IL-1β levels alone had limited success in differentiating disease status. IL-1β is present in the serum and ascites of OC patients [9] and has been shown to be involved with cancer tumorigenesis, angiogenesis, and metastasis [18]. The inability of urinary IL-1β to differentiate between benign and malignancy may be confounded by the inflammatory nature of so many benign and cancer conditions. That is, many benign ovarian conditions develop in an inflammatory microenvironment. For instance, endometrioma, a form of endometriosis in the ovary, is a highly inflammatory condition [19] which would expectedly result in high levels of urinary IL-1β [20]. Proinflammatory markers including serum C-reactive protein, IL-6, and IL-8 have all been used in clinical studies in an attempt to differentiate between normal, benign tumor, and OC [21-23]. Immunohistochemical analyses have shown differential expression of IL-18 and its receptors in benign ovarian tumors, borderline ovarian tumors, and ovarian carcinomas [24]. Therefore, developing and employing a panel of inflammatory mediators, including urinary IL-1β, may eventually benefit differential diagnostic and prognostic outcomes of OC.

Patient samples with confirmed family history of cancer were limited in this small pilot study. Nonetheless, urinary IL-1β levels tended to be highest in patients with benign ovarian disease and a (first degree) family history of breast and/or ovarian cancer compared to patients with benign ovarian disease, but without a family history of breast and/or ovarian cancer. Likewise, elevated levels of urinary IL-1β were found among OC patients with a family history of breast and/or ovarian cancer compared to OC patients from families without a family history of disease. This supports the recent dualistic model that epithelial OCs arise as either two types: Type 1 and Type 2 [25]. Type 1 tumors may arise in a step-wise progression from a benign precursor lesion such as, the highly inflammatory condition, endometriosis. However, it is important to remember that not all individuals at risk for OC develop the disease so that secondary events, perhaps beyond a family history, may be necessary to promote disease.

Obesity as a risk factor of OC remains controversial. Recently, a meta-analysis of 47 epidemiological studies found increased OC risk with high BMI [26]. The Ovarian Cancer Association Consortium investigated 15 case–control studies and found overweight and obese women were associated with increased risk of OC [27]. The National Institutes of Health also found that BMI was significantly associated with increased OC risk in women who never used hormone therapy [28]. Canchola, et al. found a positive association between OC risk and adult weight gain, waist circumference, and waist-to-hip ratio, but no association to overall obesity as classified by BMI [29]. In agreement, Delort, et. al. also noted high waist-to-hip ratio associated with increased risk of OC though they found no association with BMI [30]. In contrast, Schouten, et al. reported no overall association between BMI and risk of OC; however, they did report a positive association with high BMI and increased OC risk among premenopausal women [31]. More recent prospective studies reported no significant relationship between BMI and OC risk, irrespective of menopausal status [32,33]. Likewise, evidence reported by McGee, et. al. does not support a risk for OC with weight or weight gain among *BRCA1* or *BRCA2* mutation carriers [34]. Interestingly, Engleland et al. also reported that the risk for OC was not associated with adult BMI, but suggested a possible increased risk in women who were obese in young adulthood [35].

In this pilot study, one of the most apparent clinical features related to elevated urinary IL-1β was BMI. We found increased urinary IL-1β levels associated with higher BMI. Overweight and obese patients were most likely to be diagnosed with OC and ovarian benign disorders, respectively, while concomitantly demonstrating the highest average urinary IL-1β levels. In contrast, healthy controls with normal BMI exhibited the lowest average urinary IL-1β. Among our data was a single patient case classified as underweight, but with elevated urinary IL-1β levels and a diagnosis of OC. It is tempting to speculate that this individual may have had cachexia at the time of sample collection where weight loss due to the increased glucose, lipid, and protein requirements of the tumor [36] could manifest as low BMI compounded with elevated urinary IL-1β as a result of advanced disease.

Obesity and elevated IL-1β levels in OC patients may contribute to OC mortality. While some studies have shown no association between BMI five to ten years prior to OC diagnosis and OC mortality [37,38], they do suggest that obesity is associated with poor outcome [39]. Consequently, obesity itself may not be the leading factor for increased OC mortality, but may act as a comorbidity factor. For instance, difficulty of proper chemotherapy dosages for obese patients may contribute to poor disease outcome. A study of dosing practices of clinicians found that a significant proportion of OC patients with advanced disease were overweight or obese, as seen in the current study, and under-dosing in obese populations was common [40]. The variability in dosing when prescribing chemotherapy is largely due to concern for potential over-dosing and chemotherapy associated toxicities [41]. High mobility group A2 (HMGA2) is a protein that can regulate transcription by altering chromatin architecture and facilitate the assembly of multiprotein complexes of transcriptional factors [42]. It is overexpressed in serous OC tumors, but not in normal ovarian epithelial cells [43]. OCs with high expression of HMGA2 and high BMI negatively affected overall survival [44].

Lastly, obesity may increase tumor aggressiveness. Increased metabolic activity and glucose concentrations driven by the Warburg effect [45] are associated with highly aggressive OC cell lines [46]. In cancer, the Warburg effect is regarded as a characteristic metabolic process that may contribute to cell survival in a stressful environment, such as the stress of chronic inflammation [47]. The Warburg effect suggests that cancer cells produce energy predominately by glycolysis and lactic acid production over oxidative phosphorylation [45]. An in vivo obese mouse model demonstrated increased tumor size and tumors in these obese mice had a unique molecular makeup noted by upregulation of inflammation genes [48]. Obesity in OC patients may further exacerbate disease by contributing to an inflammatory environment. Obesity-related type 2 diabetes is associated with chronic inflammation [49-51] and IL-1β levels have been shown to be correlated with obesity and obesity related disorders. Individuals with combined elevated plasma levels of IL-1β and IL-6 are at increased risk for developing type 2 diabetes [52], but even mild weight loss in obese patients resulted in a 45% decrease in serum IL-1β levels over a three-year study period [53]. Leptin, an adipocytokine involved in the pathogenesis of insulin resistance necessary for developing type 2 diabetes, induces β-cell apoptosis and impaired β-cell function by promoting IL-1β production in human pancreatic islets [54]. Expression of leptin is also positively correlated with BMI [55]. In the current study, obese patients tended to have the highest urinary IL-1β levels and increased urinary IL-1β may be indicative of advanced disease. Consequently, urinary IL-1β levels/BMI may prove to be useful prognostic indicator of gynecologic disease.

The greatest limitation of urinary IL-1β as a biomarker for OC is kidney function. Inflammatory mediators, including IL-1β, are typically found elevated in the urine and serum of patients with impaired kidney function [56-58]. Furthermore, elevated levels of IL-1β have been reported in vaginal secretions associated with gynecologic infections; however, Basso, et al. were unable to detect IL-1β in patient urine or serum [59]. Interestingly, while the pH of our urine samples were within neutral range suggesting the absence of urinary tract infections and normal renal function, two samples in our study, 3119–2 and 3119–13, displayed unusually elevated urinary IL-1β levels. Unfortunately, clinical information pertaining to kidney injury/dysfunction or gynecological infection was unavailable as it may have contributed to excessive urinary levels of IL-1β. Clearly, the potential for confounding clinical parameters to influence the impact of urinary IL-1β levels for gynecologic disease warrants further investigation.

The data in this study are derived from a small pilot study, but is representative of our institutional clinical practice in regard to OC histology and stage distribution as well as published pilot studies examining IL-1β in urine, serum, and plasma [17,56,58-61] and other clinically relevant pilot studies [62-66]. A normal baseline value for urinary IL-1β in women has not yet been established in the literature [67]. Therefore, further study with increased sample size may assist in the development of statistically significant baseline and threshold values that could be used to differentiate between healthy, benign disorders, and OC, as well as other clinical parameters such as metabolic disruption. Further, urinary levels of IL-1β levels/BMI as a prognostic indicator of gynecologic disease could impact clinical practice.

## Conclusions

Overall, our pilot study suggests that elevated urinary IL-1β may be associated with cancer progression so that the identification of an inflammatory profile specific to epithelial OC may benefit non-invasive diagnostic and prognostic applications as well as lead to the development of adjuvant therapies utilizing target-specific anti-inflammatory treatments to reduce the mortality associated with this disease.

## Abbreviations

BMI: Body mass index
EDTA: Ethylenediaminetetraacetic acid
ELISA: Enzyme linked immunoassay
FIGO: International Federation of Gynecology and Obstetrics
HMGA2: High mobility group A2
IL-1β: Interleukin-1β
MCC: H. Lee Moffitt Cancer Center
OC: Ovarian cancer
SD: Standard deviation
SE: Standard error
TVS: Transvaginal ultrasonography
USF: University of South Florida
